# Spontaneous hyphema in the setting of COVID-19 pneumonia

**DOI:** 10.1016/j.ajoc.2022.101447

**Published:** 2022-02-18

**Authors:** Joey Chiang, Lawrence Chan, Jeannette Y. Stallworth, Matilda F. Chan

**Affiliations:** a University of California, San Francisco, School of Medicine, San Francisco, CA, USA; b University of California, San Francisco, Department of Ophthalmology, San Francisco, CA, USA; c Francis I. Proctor Foundation, University of California, San Francisco, San Francisco, CA, USA

**Keywords:** COVID-19, Hyphema, Coagulopathy, Severe Acute Respiratory Syndrome Coronavirus 2, SARS-CoV-2/COVID-19, D-Dimer, DD, Intraocular pressures, IOP, Acute myeloid leukemia, AML

## Abstract

**Purpose:**

To report a challenging case of spontaneous hyphema in the setting of prone positioning for COVID-19 pneumonia.

**Observations:**

A previously healthy patient was concomitantly diagnosed with acute myelogenous leukemia (AML) and COVID-19 infection. During his hospitalization he required intubation and prone positioning. Following change from prone to supine positioning, he was noted to have developed a large unilateral spontaneous hyphema.

**Conclusions and Importance:**

We present a challenging case of spontaneous hyphema due to a hematologic malignancy in the setting of prone positioning for COVID-19 pneumonia.

## 1 Introduction

Severe Acute Respiratory Syndrome Coronavirus 2 (SARS-CoV-2/COVID-19) has increasingly been linked to hematologic conditions such as coagulopathy and thrombotic disease in addition to its primary respiratory pathology.^1^ The first described coagulopathies are a marked rise in D-Dimer (DD) and rather moderate thrombocytopenia.^1^

Acute myeloid leukemia (AML) is a hematologic neoplasm that arises from the myeloid cell line. AML is the most common form of leukemia in adults and accounts for nearly 40% of cases. Affected individuals often present with anemia, leukopenia and thrombocytopenia. The retina is the most common site of ocular manifestations and findings include venous dilation and tortuosity, retinal hemorrhages at all levels, and cotton wool spots.^2^ The anterior segment is a rare location for leukemic involvement, but manifestations include comma-shaped venous abnormalities of the conjunctiva, conjunctival tumors, iris infiltration, and spontaneous hyphema.^2^

We report the challenging case of a patient with new concomitant diagnoses of AML and COVID-19 infection requiring intubation and prone positioning who developed unilateral spontaneous hyphema. To our knowledge, this is the first reported case of spontaneous hyphema in the setting of COVID-19 pneumonia.

## 2 Case report

A 37-year-old previously healthy male presented to the emergency room with abdominal pain, bone pain, and leukocytosis. A bone marrow biopsy was performed and revealed AML. The patient tested positive for COVID-19 but was initially asymptomatic. However, over the following days, he subsequently became hypoxic and required intubation with prone positioning as well as treatment with Remdesivir, dexamethasone, and convalescent plasma therapy.

**Fig. 1:**
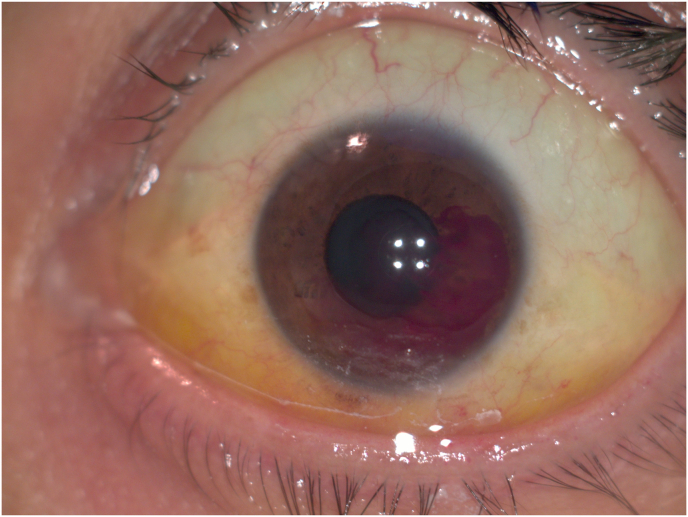
External photograph of left eye. Ointment and epitheliopathy are present at the inferior peripheral cornea. There are clots forming inferotemporally within the hyphema.

**Fig. 2:**
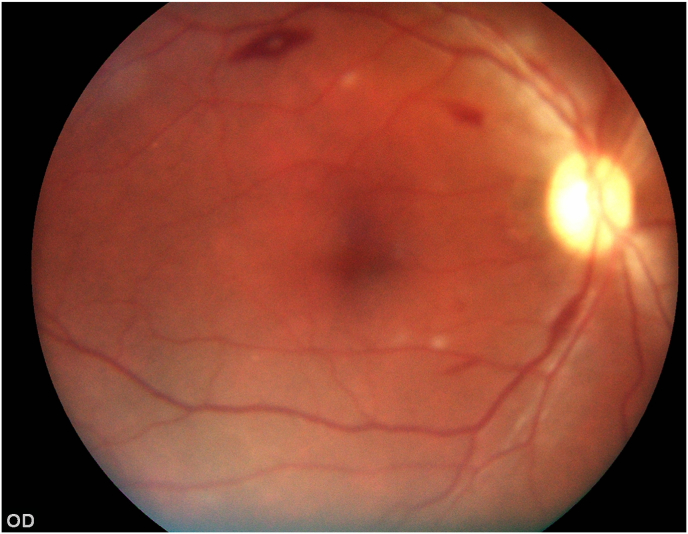
Fundus photo of the right eye. There were scattered flame hemorrhages, cotton-wool spots, and a white-centered hemorrhage at the superior arcade.

The ophthalmology service was consulted after the patient was noted to have a hyphema of the left eye following change from prone to supine positioning. There was no known ocular trauma. Visual acuity was not assessed as the patient was intubated and sedated. Intraocular pressures (IOP) were 10 mmHg OD and 12 mmHg OS, although subsequently became elevated to 26 mmHg OS. Portable slit lamp exam was notable for a hyphema OS at 2 mm height with inferotemporal clots (Fig. 1). Dilated fundus exam revealed scattered flame hemorrhages, cotton-wool spots, and Roth spots OD (Fig. 2); OS fundus view was limited by the hyphema.

Atropine and IOP-lowering drops were started, in addition to head of bed elevation and placement of a rigid eye shield. The appearance of the hyphema remained stable on serial follow-up exams. The patient deteriorated four days after initial evaluation with worsening hypoxemic respiratory failure and septic shock. He died shortly after due to cardiac arrest.

## 3 Discussion

We present the case of a previously healthy patient with newly diagnosed AML who additionally developed severe COVID-19 pneumonia, and was found to have a spontaneous hyphema in the setting of prone positioning and marked thrombocytopenia. While previous reports of ophthalmic manifestations associated with COVID-19 have been limited to findings associated with viral conjunctivitis such as epiphora, chemosis, and conjunctival hyperemia, there have been no reports of spontaneous hyphema in the setting of a COVID-19 infection.^3^ Hyphema most-commonly develops in the setting of recent trauma; however, rare cases can occur spontaneously in the setting of inflammatory or hematologic disease.^4^^,^^5^ A confluence of factors likely contributed to our patient's spontaneous hyphema: 1) AML causing pancytopenia with profound thrombocytopenia (6 × 10^9^/L); 2) prolonged prone positioning resulting in increased episcleral venous pressure, possibly with unnoticed blunt trauma or compression of the eye; and 3) bleeding diathesis from COVID-related thrombocytopenia. While spontaneous hyphemas have been observed with sudden increases in intrathoracic or abdominal pressures,^6^^,^^7^ our patient's intubation and pulmonary status likely did not have a significant role in the development of his hyphema. Although intubation can lead to hemodynamic changes, our patient had been intubated for three days prior to when the hyphema was first noticed. Additionally, his blood pressure was low and he was on vasopressors to maintain systemic perfusion. Prolonged prone positioning is a commonly utilized strategy to improve oxygenation in the critical care setting, and has been associated with various ocular complications such as corneal abrasion, ischemic neuropathy, retinal artery occlusion, and orbital compartment syndrome.^8^^,^^9^ The need for prone positioning for our patient likely inhibited the resolution of his hyphema.

## 4 Conclusions

In summary, this report adds spontaneous hyphema to the clinical spectrum of ocular findings associated with COVID-19 infection. We describe a patient with new concomitant diagnoses of AML and COVID-19 infection who developed a spontaneous hyphema following prone positioning. This case highlights the potential hematologic complications of COVID-19 and its associated treatment. It additionally underscores the importance of ophthalmic surveillance in critically ill patients with COVID-19 to identify early signs of ophthalmic pathology in order to initiate treatment promptly.

## Funding

Research to Prevent Blindness Unrestricted Grant to the 10.13039/100008069UCSF Department of Ophthalmology and All May See Foundation.

## Authorship

All authors attest that they meet the current ICMJE criteria for Authorship.

## Patient consent

Consent to publish the case report was not obtained. This report does not contain any personal information that could lead to the identification of the patient.

## Declaration of competing interest

The following authors have no financial disclosures: J.C., L.C., J.Y.S, M.F.C.
